# KMT2D Deficiency Promotes Myeloid Leukemias which Is Vulnerable to Ribosome Biogenesis Inhibition

**DOI:** 10.1002/advs.202206098

**Published:** 2023-05-04

**Authors:** Jing Xu, Ailing Zhong, Shan Zhang, Mei Chen, Lanxin Zhang, Xiaohang Hang, Jianan Zheng, Baohong Wu, Xintong Deng, Xiangyu Pan, Zhongwang Wang, Lu Qi, Kaidou Shi, Shujun Li, Yiyun Wang, Manli Wang, Xuelan Chen, Qi Zhang, Pengpeng Liu, Robert Peter Gale, Chong Chen, Yu Liu, Ting Niu

**Affiliations:** ^1^ Department of Hematology Institute of Hematology State Key Laboratory of Biotherapy and Cancer Center West China Hospital Sichuan University Chengdu 610041 China; ^2^ Centre for Hematology Imperial College of Science Technology and Medicine London SW7 2BX UK; ^3^ Department of Hematologic Oncology Sun Yat‐sen Cancer Center Guangzhou 510060 China

**Keywords:** acute myeloid leukemia, epigenetics, KMT2D, mTOR, ribosome biogenesis

## Abstract

*KMT2C and KMT2D* are the most frequently mutated epigenetic genes in human cancers. While *KMT2C* is identified as a tumor suppressor in acute myeloid leukemia (AML), the role of *KMT2D* remains unclear in this disease, though its loss promotes B cell lymphoma and various solid cancers. Here, it is reported that *KMT2D* is downregulated or mutated in AML and its deficiency, through shRNA knockdown or CRISPR/Cas9 editing, accelerates leukemogenesis in mice. Hematopoietic stem and progenitor cells and AML cells with *Kmt2d* loss have significantly enhanced ribosome biogenesis and consistently, enlarged nucleolus, increased rRNA and protein synthesis rates. Mechanistically, it is found that *KMT2D* deficiency leads to the activation of the mTOR pathway in both mouse and human AML cells. *Kmt2d* directly regulates the expression of *Ddit4*, a negative regulator of the mTOR pathway. Consistent with the abnormal ribosome biogenesis, it is shown that CX‐5461, an inhibitor of RNA polymerase I, significantly restrains the growth of AML with *Kmt2d* loss in vivo and extends the survival of leukemic mice. These studies validate *KMT2D* as a de facto tumor suppressor in AML and reveal an unprecedented vulnerability to ribosome biogenesis inhibition.

## Graphical abstract

*KMT2D* is identified as a tumor suppressor gene in acute myeloid leukemia. *KMT2D* deficiency reduces H3K4 methylation levels and suppresses the expression of *DDIT4*, the negative regulator in the mTOR signaling pathway, which leads to the activation of the mTOR pathway and enhancement of ribosome biogenesis. *KMT2D*‐deficient cells are vulnerable to ribosome biogenesis inhibitors such as CX‐5461.

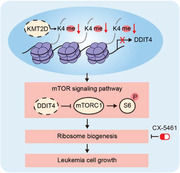

## 1 Introduction

Epigenetic factors are among the most frequently altered genes in human cancers.^[^ ^1^ ^]^ In acute myeloid leukemia (AML), there is accumulating evidence indicating that mutations of epigenetic regulators, including writers, readers, and erasers, play significant roles in the disease initiation, progression, and drug response.^[^ ^2^, ^3^ ^]^ Among them, the lysine methyltransferase 2 (*KMT2*, also known as mixed‐lineage leukemia (*MLL*)) family genes are arguably the most notable in AML.^[^ ^4^ ^]^ There are five *KMT2* family members, *KMT2A‐E*, given their functions to methylate the lysine residues of histones.^[^ ^5^ ^]^ *KMT2A* is the major player in large MLL‐fusion proteins found in AML and acute lymphoblastic leukemia, which are often associated with poor prognosis.^[^ ^6^ ^]^ *MLL*‐fusion genes have been demonstrated as driver genes to promote AML genesis in mice.^[^ ^7^ ^]^ In contrast, *KMT2C*, located on chromosome 7q36.1, is recurrently deleted in −7/del7q AML. The heterozygous loss of *KMT2C* could promote AML formation in mice.^[^ ^8^ ^]^ KMT2C and KMT2D are large scaffold proteins that form the KMT2C/D COMPASS complex (complex of proteins associated with Set1), which contains WDR5, RBBP5, hDPY30, ASH2, KDM6A (UTX), PTIP, PA1, and NCOA6.^[^ ^9^ ^]^ Besides *KMT2C*, it is also reported that loss‐of‐function mutations of *KDM6A*, which happened in AML, promoted myeloid leukemogenesis and contributed to chemotherapy resistance.^[^ ^10^, ^11^ ^]^

*KMT2D*, a histone‐3 lysine‐4 methyltransferase, is one of the most frequently mutated genes like *KMT2C* in human cancers.^[^ ^12^, ^13^, ^14^ ^]^ *KMT2D* plays critical roles in various biological processes, including regulation of development, differentiation, metabolism, and tumor suppression, while its frequent mutations have been found in developmental diseases, such as Kabuki syndrome and congenital heart disease, and various forms of cancers.^[^ ^12^ ^]^ In many solid cancers and B‐cell lymphomas, *KMT2D* deficiency or its loss‐of‐function mutants drive the initiation and development of tumors.^[^ ^15^, ^16^, ^17^, ^18^, ^19^, ^20^, ^21^ ^]^ Its deficiency induces wide epigenetic alterations that affect multiple cellular functions, including transcription stress, genomic stability, metabolism reprogramming, and response to immune checkpoint blockade.^[^ ^17^, ^22^, ^23^, ^24^ ^]^ *KMT2D* is frequently mutated in a cohort of Chinese AML.^[^ ^25^ ^]^ However, several studies in *MLL‐AF9*‐ or *HOXA9*‐driven AML found that *Kmt2d* deficiency did not drive but suppress leukemia formation, which seems to be the opposite effect of *Kmt2d* on other cancers like B‐cell lymphoma.^[^ ^26^, ^27^ ^]^ Therefore, it is worthwhile to investigate the function and mechanism of *KMT2D* on leukemia genesis and maintenance.

Given the frequent, seemly loss‐of‐function mutations of *KMT2D* in AML and its tumor suppression capacity in other cancers, we hypothesized *KMT2D* is a tumor suppressor in AML. To test our hypothesis, we studied a mouse AML model where we reduced *Kmt2d* expression by shRNA knockdown and CRISPR/Cas9 technology. Further, we found that ribosome biogenesis was dramatically increased in *Kmt2d*‐deficient hematopoietic stem and progenitor cells (HSPCs) and AML cells. Inhibition of rRNA synthesis could significantly prolong the survival of *Kmt2d*‐deficient AML mice. Altogether, our data indicate that *Kmt2d* deficiency could promote AML and confer a new vulnerability to ribosome biogenesis inhibitors.

## 2 Results

### 2.1 *Kmt2d* Downregulation Promotes Acute Myeloid Leukemogenesis

**Figure 1:**
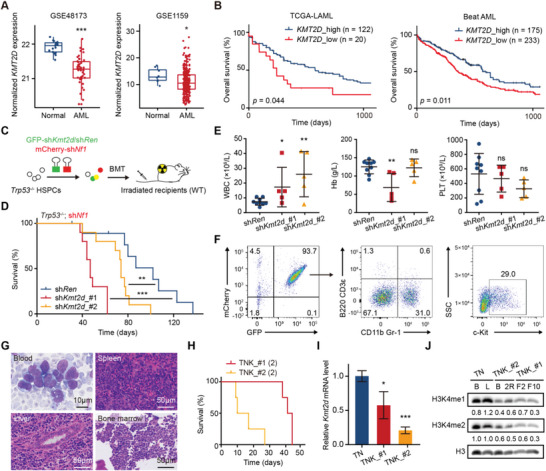
*Kmt2d* deficiency by shRNAs promotes AML in mice. A) Expression levels of *KMT2D* in AML and normal samples. Left, data were analyzed from RNA‐seq data (GSE48173) with 17 CD34^+^ cord blood and 43 AML samples; Right, data were analyzed from microarray data (GSE1159) with eight healthy donors (five normal bone marrow and three CD34^+^ cell samples) and 285 AML samples; *p < 0.05, ***p < 0.001 (two‐tailed Wilcoxon rank‐sum test). B) Survival curves of AML patients stratified by high and low expression of *KMT2D* in the TCGA‐LAML (left) and Beat AML (right) cohort, respectively. The cut‐off values were determined by maximally selected rank statistics. *p* Values were determined by the log‐rank test. C) Schematic experimental design for mouse modeling using shRNA technique. *Trp53* ^−/−^ mouse HSPCs were transduced with GFP‐linked sh*Kmt2d*/sh*Ren* and mCherry‐linked sh*Nf1*, and then transplanted into sub‐lethally irradiated syngeneic mice. D) Kaplan–Meier survival curves of mice transplanted with *Trp53* ^−/−^ HSPCs transduced with sh*Nf1* and sh*Ren* (blue; *n* = 10), sh*Kmt2d_#*1 (red; *n* = 10), or sh*Kmt2d_#*2 (orange; *n* = 10). **p < 0.01, ***p < 0.001 (log‐rank test). The results were the combination of two independent trials. E) White blood cell (WBC), hemoglobin (Hb), and platelet (PLT) counts of sh*Kmt2d* and sh*Ren* mice 7 weeks post‐transplant. F) Representative flow cytometric profiles showing the expression of fluorescent markers (GFP and mCherry), myeloid lineage markers (CD11b and Gr‐1), lymphoid lineage markers (B220 and CD3ε), and stem cell marker (c‐Kit) in bone marrow cells of sacrificed TNK (*Trp53* ^−/−^; sh*Nf1*; sh*Kmt2d*) mice. G) Representative images of histological analyses of blood, spleen, liver, and bone marrow of sacrificed TNK mice. H) Kaplan–Meier survival curves of secondary transplants from two independent primary leukemia cells of each TNK mouse (*n* = 4 per group). I) Relative mRNA levels of *Kmt2d* in bone marrow cells of sacrificed control TN (*Trp53* ^−/−^; sh*Nf1*; sh*Ren*) and TNK mice were quantified by qRT‐PCR (normalized to *Actin*; *n* = 3 per group). J) Western blotting analyses showing the H3K4me1 and H3K4me2 levels in bone marrow cell lysates from sacrificed TN and TNK mice. E,I) Graph represents the mean ± SD; *p < 0.05, **p < 0.01, ***p < 0.001, ns, not significant (unpaired two‐tailed *t*‐test).

Investigating the expression profiles of *KMT2D* in AML patients, we found that compared to normal control (CD34^+^ cord blood samples, *n* = 17), AML samples (*n* = 43) contained significantly lower *KMT2D* expression (GSE48173^[^ ^28^ ^]^). A similar observation was obtained from another cohort (GSE1159:^[^ ^29^ ^]^ 285 AML samples vs five normal bone marrow and three CD34^+^ cell samples; **Figure** **1**A and Table S1, Supporting Information). Moreover, AML patients with lower *KMT2D* expression levels were associated with shorter overall survival, according to the analysis from the TCGA^[^ ^4^ ^]^ or Beat^[^ ^30^ ^]^ AML cohort (Figure 1B and Table S1, Supporting Information). These seemingly generally reduced expressions of *KMT2D* in AML suggest that *KMT2D* deficiency may contribute to AML development.

To investigate the role of *KMT2D* in leukemogenesis, we developed a transplantation‐based mouse model with RNA interference approach. Both shRNAs effectively repressing *Kmt2d* (sh*Kmt2d*) in mouse cells were cloned and confirmed by quantitative real‐time polymerase chain reaction (qRT‐PCR, Figure S1A, Supporting Information). Since *KMT2D* mutations in patients with leukemia co‐occur with *TP53* and *NF1* mutations, we tested the effects of *Kmt2d* deficiency in mice in the context of *Trp53* and *Nf1* deletion (Figure S1B and Table S1, Supporting Information). We transplanted c‐Kit^+^ HSPCs infected with GFP‐linked sh*Kmt2d* or control *Renilla* shRNA (sh*Ren*), together with *Trp53* and *Nf1* loss, into sub‐lethally irradiated syngeneic wild‐type C57BL/6 recipient mice (Figure 1C and Figure S1C, Supporting Information). After transplantation, recipient mice were monitored weekly. Compared to control recipient mice, mice transplanted with *Trp53* ^−/−^; sh*Nf1*; sh*Kmt2d* HSPCs (hereafter referred to as TNK) developed AML significantly faster with shorter overall survival (sh*Kmt2d*_#1: median 47 days after transplantation; *p* = 0.0004 and sh*Kmt2d*_#2: median 74 days after transplantation; *p* = 0.0094; Figure 1D).

Recipients with sh*Kmt2d* HSPCs displayed significantly increased peripheral white blood cell (WBC) counts compared to controls 7 weeks after transplantation, while the former had variably decreased hemoglobin (Hb) and platelet (PLT) levels, indicative of leukemic outgrowth with the suppression of normal hematopoiesis (Figure 1E). Flow cytometry results showed that GFP‐linked sh*Kmt2d* was enriched in leukemia cells, indicating a selective advantage of cells with *Kmt2d* knockdown during leukemia development (Figure 1F). All recipients with *Kmt2d* knockdown developed AML with neoplastic cells expressing stem and progenitor marker c‐Kit as well as myeloid surface markers CD11b/Gr‐1 (Figure 1F), and peripheral blood smears showing leukocytosis with increased numbers of neutrophils, monocytes, and blasts, except three mice in one hairpin sh*Kmt2d* (sh*Kmt2d*_#2) who had mixed lineage leukemia (Figure 1G). Sacrificed mice showed significant hepatomegaly (mean liver weight, sh*Kmt2d*_#1: 3.387 g; sh*Kmt2d*_#2: 2.116 g) and splenomegaly (mean spleen weight, sh*Kmt2d*_#1: 1.187 g; sh*Kmt2d*_#2: 0.618 g) because of extramedullary hematopoiesis and leukemia infiltration. Hematoxylin and eosin (H&E) staining of the spleen, liver, and bone marrow revealed prominent leukemia with the disruption of normal architecture (Figure 1G). Harvested bone marrow cells enriched with leukemia cells could generate AML in secondary recipient mice (Figure 1H and Figure S1D,E, Supporting Information). Control sh*Ren* recipients developed AML eventually with similar immunophenotype and histopathology, despite a longer tumor formation time.

We confirmed that AML generated above is driven by *Kmt2d* suppression. First, *Kmt2d* mRNA levels in harvested leukemia cells were dramatically reduced (Figure 1I). Then, since KMT2D is a writer of H3K4 mono‐ and di‐methylation, we also noticed the consistent reduction of H3K4me1 and H3K4me2 levels in leukemia cells with *Kmt2d* knockdown, indicating on‐target effects of sh*Kmt2d* (Figure 1J). Hence, our results demonstrated that suppression of *Kmt2d* promotes AML genesis in mice.

### 2.2 *Kmt2d* Loss‐of‐Function Mutants Drive AML As Well

**Figure 2:**
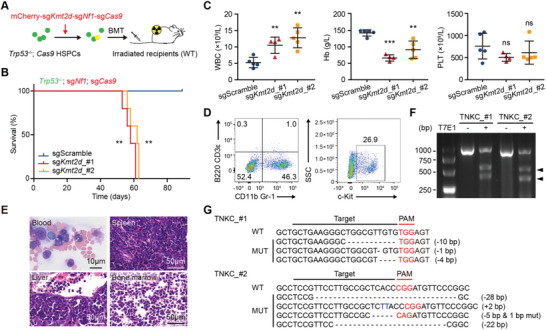
*Kmt2d* mutation by CRISPR/Cas9 promotes AML in mice. A) Schematic experimental design for mouse modeling using CRISPR/Cas9 system. *Trp53* ^−/−^; *Cas9* mouse HSPCs were transduced with mCherry‐linked sg*Kmt2d*‐sg*Nf1*‐sg*Cas9* or sgScramble‐sg*Nf1*‐sg*Cas9*, and then transplanted into sub‐lethally irradiated syngeneic mice. B) Kaplan–Meier survival curves of mice transplanted with *Trp53* ^−/−^; *Cas9* HSPCs transduced with sgScramble‐sg*Nf1*‐sg*Cas9* (blue; *n* = 5), sg*Kmt2d_*#1‐sg*Nf1*‐sg*Cas9* (red, *n* = 5), or sg*Kmt2d_#*2‐sg*Nf1*‐sg*Cas9* (orange; *n* = 5). **p < 0.01 (log‐rank test). C) WBC, Hb, and PLT counts of sgScramble and sg*Kmt2d* mice 2 months post‐transplant. Graph represents the mean ± SD; **p < 0.01, ***p < 0.001, ns, not significant (unpaired two‐tailed *t*‐test). D) Representative flow cytometric profiles showing the expression of CD11b/Gr‐1, B220/CD3ε and c‐Kit in bone marrow cells of sacrificed TNKC (*Trp53* ^−/−^; sg*Nf1*; sg*Kmt2d;* sg*Cas9*) mice. E) Representative images of histological analyses of blood, spleen, liver, and bone marrow of sacrificed TNKC mice. F) T7 endonuclease I assay on *Kmt2d* in bone marrow cells of sacrificed TNKC mice. G) Mutation analyses of the *Kmt2d* regions targeted by CRISPR/Cas9 of TNKC bone marrow cells. Representative Sanger sequences of single TA clones.

We then analyzed the status of *KMT2D* alteration in AML patients (data from cBioProtal). About 1.6% (3/190) of TCGA AMLs carry *KMT2D* mutants or deletions.^[^ ^4^ ^]^ Interestingly, in the OHSU AML cohort (no copy number alteration data), *KMT2D* mutations (5/531) are all truncating mutations^[^ ^30^ ^]^ (Figure S2A and Table S1, Supporting Information), suggesting a loss‐of‐function mechanism of *KMT2D* in AML. Thus, we further applied CRISPR/Cas9 genome editing technique to generate *Kmt2d* mutants in HSPCs and studied their effects on AML development. One of two independent mCherry‐labeled tandem single guide RNAs (sgRNAs) targeting the genomic DNA sequence encoding the PHD domain of *Kmt2d (*sg*Kmt2d)*, together with *Nf1* sgRNA (sg*Nf1*), were transduced into c‐Kit^+^ HSPCs from *Trp53* ^−/−^; *Cas9* knock‐in transgenic mice, followed by transplantation into 5.5 Gy irradiated syngeneic wild‐type mice (**Figure** **2**A). A sgRNA targeting *Cas9* (sg*Cas9*) was also used to prevent potential adverse effects associated with constitutive *Cas9* expression.

Recipients carrying *Trp53* ^−/−^; sg*Nf1*; sg*Kmt2d;* sg*Cas9* HSPCs (hereafter referred to as TNKC) died of AML at a median survival of 59 days, while none of the control sgScramble recipients developed diseases during the observation period (Figure 2B). Consistent with sh*Kmt2d* AML developed above, *Kmt2d* mutants accelerated AML development evidenced by leukocytosis, anemia, and thrombocytopenia in recipients of sg*Kmt2d* HSPCs compared to controls 2 months post‐transplant (Figure 2C). Harvested bone marrow cells from moribund sg*Kmt2d* mice were CD11b/Gr‐1^+^ and c‐Kit^+^ (Figure 2D). Blood smear and H&E staining of the liver, spleen, and bone marrow showed accumulated blasts and aggressive leukemia cell infiltration (Figure 2E). In the harvested leukemia cells, *Kmt2d* mutations were confirmed by T7 endonuclease I mismatch detection assay and sanger sequences (Figure 2F,G). Of note, all *Kmt2d* detected mutations were truncating, supporting a loss‐of‐function mechanism of *Kmt2d* in AML.

### 2.3 *Kmt2d* Deficiency Upregulates Ribosome Biogenesis

**Figure 3:**
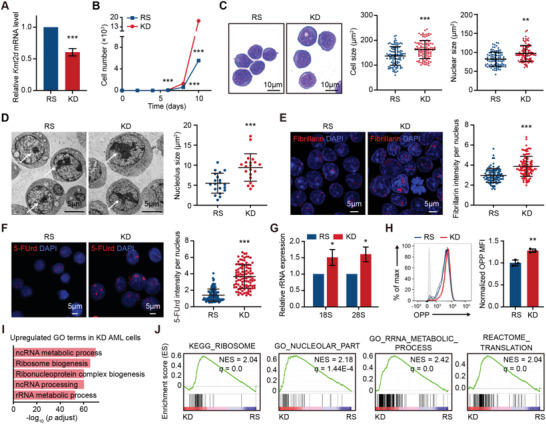
*Kmt2d* negatively regulates ribosome biogenesis in AML. A) Relative mRNA levels of *Kmt2d* in AML cells obtained from TRE‐rtTA‐driven inducible *Kmt2d* knockdown mice treated with doxycycline (sh*Kmt2d*, KD) or without doxycycline (*Kmt2d*‐restored, RS) were quantified by qRT‐PCR (normalized to *Hprt*; *n* = 3 per group). B) The effect of *Kmt2d* knockdown on cell growth (*n* = 3 per group). C) The effect of *Kmt2d* knockdown on cell morphology. Representative pictures performed on Liu's‐stained cytospins (left) and quantitation of cell size and nuclear size (right) in *Kmt2d* restored and knockdown AML cells (*n* = 100 per group). D) The effect of *Kmt2d* knockdown on nucleolus size. Representative transmission electron microscopy images (left) and quantitation of nucleolus size (right) in *Kmt2d* restored and knockdown AML cells (*n* = 20 per group). E) The effect of *Kmt2d* knockdown on the intensity of nucleolar protein fibrillarin (red). Representative immunofluorescence images (left) and quantitation of fluorescence intensity per nucleus (right) in *Kmt2d* restored and knockdown AML cells (*n* = 100 per group). F) The effect of *Kmt2d* knockdown on rRNA synthesis. Cells were labeled with 5‐FUrd for 30 min and immunostained with an antibody against BrdU. Representative immunofluorescence images (left) and quantitation of fluorescence intensity per nucleus (right) in *Kmt2d* restored and knockdown AML cells (*n* = 100 per group). G) The effect of *Kmt2d* knockdown on relative levels of 18S and 28S rRNA was quantified by qRT‐PCR in *Kmt2d* restored and knockdown AML cells (normalized to Actin; *n* = 3 per group). H) The effect of *Kmt2d* knockdown on protein synthesis was performed by OPP incorporation assay in *Kmt2d* restored and knockdown AML cells (*n* = 3 per group). Representative flow cytometric profile (left) and quantitation of OPP MFI (right) (*n* = 3 per group). I) GO analysis of significantly upregulated genes in *Kmt2d* knockdown AML cells compared to restored cells (log2‐fold change > 0.5, *p* < 0.05). *p* adjust values were determined by Benjamini–Hochberg correction (*p* adjust < 0.05). J) GSEA showing the positive enrichment of the KEGG_RIBOSOME, GO_NUCLEOLAR_PART, GO_RRNA_METABOLIC_PROCESS, and REACTOME_TRANSLATION gene sets in *Kmt2d* knockdown AML cells compared to restored cells. C–F) Quantitation was analyzed by Image J. B–H) Graph represents the mean ± SD, *p < 0.05, **p < 0.01, ***p < 0.001 (unpaired two‐tailed *t*‐test).

To investigate the role of *Kmt2d* in AML, we generated a mouse inducible sh*Kmt2d*‐driven AML with the Tet‐ON system. In this system, sh*Kmt2d* with the fluorescence gene dsRed is regulated by the tetracycline‐inducible promoter TRE. Treatment with doxycycline activates the TRE promoter resulting in the transcription of sh*Kmt2d* (*Kmt2d* knockdown, KD), while a withdraw time of 4 days shuts down the TRE promoter and sh*Kmt2d* transcription (*Kmt2d* restored, RS, Figure S3A, Supporting Information). sh*Kmt2d* AML cells presented with venus and dsRed double‐positive population by flow cytometry and the expression of *Kmt2d* was reduced, assessed by qRT‐PCR (**Figure** **3**A and Figure S3B, Supporting Information). Remarkably, repressing *Kmt2d* expression resulted in a significant growth increment of AML cells (Figure 3B). Moreover, both cell size and nuclear size were enlarged significantly in *Kmt2d*‐deficient AML cells (Figure 3C).

Since cell proliferation, cell size, and carcinogenesis have a close relationship with the upregulated ribosome biogenesis, we detected cellular ribosomal function in *Kmt2d*‐deficient AML cells.^[^ ^31^, ^32^, ^33^, ^34^ ^]^ Nucleoli are the sites to produce and assemble ribosomes. Studies have shown the connection between structural and functional alterations of nucleoli and tumorigenesis, where increased ribosome biogenesis is associated with larger nucleoli and malignant tumors generally have larger ones.^[^ ^35^, ^36^ ^]^ From the analysis of transmission electron microscopic (TEM) images and immunofluorescence staining using an antibody against nucleolar protein fibrillarin, sh*Kmt2d* AML cells contained significantly enlarged nucleoli and brighter ribosome biogenesis key molecule fibrillarin staining, compared to control cells (Figure 3D,E). Ribosomes are comprised of ribosomal proteins and ribosomal RNAs (rRNAs). We evaluated the rRNA synthesis by the immunofluorescence detection of 5‐fluorouridine (5‐FUrd) incorporation into nascent rRNAs. Cells were pulse‐labeled for 30 min with 5‐FUrd and immunostained with an antibody against BrdU. Results showed that *Kmt2d* deficiency greatly enhanced rRNA transcription (Figure 3F). Further, we estimated the total rRNA concentration by nondenaturing agarose gel electrophoresis and observed that rRNA levels were elevated when *Kmt2d* knockdown (Figure S3C, Supporting Information). Quantitatively, both 18S and 28S rRNA transcription levels were increased in *Kmt2d* knockdown AML cells, compared to *Kmt2d* restored cells (Figure 3G). To investigate the function of increased ribosomes, we measured the newly synthesized peptides with O‐propargyl‐puromycin (OPP) protein synthesis assays. Briefly, OPP, a membrane‐permeable puromycin analog, was added to leukemia cells and then measured by flow cytometry. OPP exerts its inhibition by incorporating into nascent peptides, disrupting peptides transfer on ribosomes, and causing premature chain termination during translation. As a result, *Kmt2d*‐deficient AML cells contained stronger OPP signals indicating higher translational activity, compared to *Kmt2d* restored AML cells (Figure 3H). Thus, our results demonstrated that *Kmt2d* deficiency upregulates ribosome biogenesis and increases protein synthesis.

To further get insight into the alterations in ribosome biogenesis, we performed RNA sequencing (RNA‐seq) to profile the transcriptomes of *Kmt2d* knockdown versus restored AML cells. As expected, *Kmt2d* deficiency induced wide gene expression changes, with more significantly downregulated genes (945 down vs 499 up genes, absolute log2‐fold change > 0.5, *p* < 0.05), in line with the impact of H3K4 methylation on activating gene expressions (Figure S3D and Table S2, Supporting Information). Gene ontology (GO) analyses showed that downregulated genes in sh*Kmt2d* AML cells were highly enriched in pathways including hematopoietic cell differentiation, while upregulated genes were highly enriched in pathways including ribosome biogenesis, ribonucleoprotein complex biogenesis, and rRNA metabolic process (Figure 3I and Figure S3E, Supporting Information). Gene set enrichment analyses (GSEA) further confirmed the enrichment of genes implicated in the aforementioned biological processes, with the gene signature of KEGG_RIBOSOME, GO_NUCLEOLAR_PART, GO_RRNA_METABOLIC_PROCESS, and REACTOME_TRANSLATION being significantly positively enriched in *Kmt2d* knockdown AML cells compared to *Kmt2d* restored cells (Figure 3J). The upregulated expressions of several ribosome biogenesis‐related genes like *Rpp40*, *Tbl3*, and *Fbl* in sh*Kmt2d* AML cells have been validated by qRT‐PCR (Figure S3F, Supporting Information). A similar correlation between *Kmt2d* deficiency and upregulated ribosome biogenesis was observed in HSPCs (Figure S3G–P and Table S3, Supporting Information). Interestingly, the higher ribosome biogenesis correlated with *Kmt2d* deficiency was not observed in *Kmt2c*, another member of the COMPASS‐like complex, ‐deficient HSPCs (Figure S3Q–S, Supporting Information).

### 2.4 *Kmt2d* Directly Regulates *Ddit4*, Encoding a Negative Regulator of the mTOR Pathway

**Figure 4:**
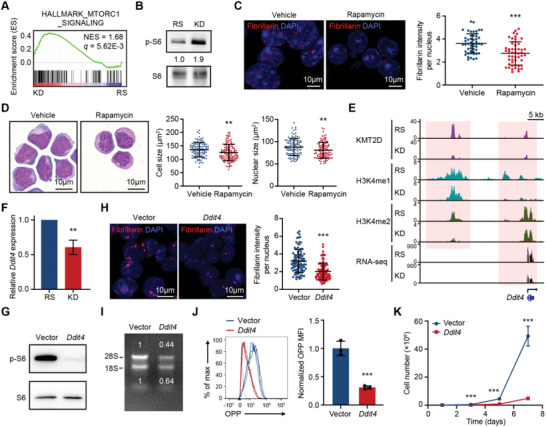
*Kmt2d* regulates ribosome biogenesis through the mTOR signaling pathway. A) GSEA showing the positive enrichment of the HALLMARK_MTORC1_SIGNALING gene set in *Kmt2d* knockdown AML cells compared to restored cells. B) Representative western blotting showing the phosphorylation levels of ribosomal protein S6 in *Kmt2d* restored and knockdown AML cells. Leukemia cells obtained from two inducible sh*Kmt2d*‐driven AML mice were tested. C) The effect of mTOR inhibitor rapamycin on the intensity of nucleolar protein fibrillarin (red). Representative immunofluorescence images (left) and quantitation of fluorescence intensity per nucleus (right) in rapamycin‐treated and vehicle‐treated *Kmt2d* knockdown AML cells (*n* = 50 per group). D) The effect of rapamycin on cell morphology. Representative pictures performed on Liu's‐stained cytospins (left) and quantitation of cell size and nuclear size (right) of rapamycin‐treated and vehicle‐treated *Kmt2d* knockdown AML cells (*n* = 100 per group). E) Representative Integrative Genomics Viewer browser tracks of KMT2D, H3K4me1, and H3K4me2 peaks on *Ddit4* locus and *Ddit4* mRNA reads in *Kmt2d* restored and knockdown AML cells. F) Relative mRNA levels of *Ddit4* in *Kmt2d* restored and knockdown AML cells were quantified by qRT‐PCR (normalized to *Hprt*, *n* = 3 per group). G) Representative western blotting showing the effect of *Ddit4* overexpression on phosphorylation levels of ribosomal protein S6 in *Kmt2d* knockdown AML cells. Leukemia cells obtained from two inducible sh*Kmt2d*‐driven AML mice were tested. H) The effect of *Ddit4* overexpression on the intensity of nucleolar protein fibrillarin (red) in *Kmt2d* knockdown AML cells. Representative immunofluorescence images (left) and quantitation of fluorescence intensity per nucleus (right) (*n* = 90 per group). I) The effect of *Ddit4* overexpression on total RNA from *Kmt2d* knockdown AML cells was separated by nondenaturing agarose gel (1%) electrophoresis. The positions of 18S and 28S ribosomal RNA are indicated. J) The effect of *Ddit4* overexpression on protein synthesis was performed by OPP incorporation assay in *Kmt2d* knockdown AML cells. Representative flow cytometric profile (left) and quantitation of OPP MFI (right) (*n* = 3 per group). K) The effect of *Ddit4* overexpression on *Kmt2d* knockdown AML cell growth (*n* = 3 per group). B,C,D,H,I) Quantitation was analyzed by ImageJ. C,D,F,H,J,K) Graph represents the mean ± SD, **p < 0.01, ***p < 0.001 (unpaired two‐tailed *t*‐test).

To explore the molecular mechanism under which *Kmt2d* regulates ribosome biogenesis, we sought to conduct multiomics analyses to identify downstream pathways and targets of *Kmt2d*. First, digging deeper into RNA‐seq data using GSEA, we found that genes in the HALLMARK_MTORC1_SIGNALING pathway were significantly positively enriched in sh*Kmt2d* leukemia cells and HSPCs (**Figure** **4**A and Figure S4A, Supporting Information). Given the role of the mammalian target of rapamycin complex 1 (mTORC1) in promoting ribosome biogenesis,^[^ ^37^, ^38^ ^]^ we hypothesized that mTOR activity may underlie *Kmt2d* deficiency‐induced ribosome upregulation. The activation of the mTOR pathway was confirmed in *Kmt2d*‐deficient leukemia cells, as shown by Western blots with antiphosphorylated ribosomal protein S6, a substrate of direct mTORC1 target S6K1 (Figure 4B). The inhibition of mTOR by rapamycin reduced the fluorescence intensity of fibrillarin staining in sh*Kmt2d* AML cells, indicating the repression of ribosome biogenesis (Figure 4C). Further, rapamycin‐treated leukemia cells were presented with smaller cell and nuclear sizes, pharmacologically reversing the effect of *Kmt2d* knockdown, further providing experimental evidence for the role of the mTOR signaling pathway played in the tumorigenesis (Figure 4D).

To assess how the mTOR signaling pathway is highly activated by *Kmt2d* deficiency and given that KMT2D, as a histone lysine N‐methyltransferase, is required for H3K4 mono‐ and di‐methylation, we performed the cleavage under targets and tagmentation (CUT&Tag) technique to investigate the corresponding H3K4me1, H3K4me2, or H3K27ac levels in *Kmt2d* restored and knockdown AML cells. Consistent with the KMT2D enzymatic activity, *Kmt2d*‐deficient AML cells had a decrease in differentially modified peak numbers of H3K4me1, H3K4me2, and H3K27ac. Specifically, in comparison with the control group, *Kmt2d*‐deficient AML cells exhibited 6680, 2592, and 1486 significantly lower H3K4me1, H3K4me2, and H3K27ac modified peaks, respectively (*p* < 0.05, log2‐fold change < −1; Figure S4B and Table S4, Supporting Information). ATAC‐seq also demonstrated a great genome accessibility change, with 1064 decreased chromatin accessibility sites in sh*Kmt2d* AML cells compared to the control group (*p* < 0.05, log2‐fold change < −0.5; Figure S4C and Table S4, Supporting Information). Remarkably, despite *Kmt2d* deficiency reprogrammed epigenetic landscape in AML cells with decreases in differentially modified peak numbers and average signal levels of gene‐activating mark H3K27ac as well as chromatin accessibility globally, RNA polymerase I (Pol I) and RNA polymerase III (Pol III) bound regions, which specialize in the transcription of rRNA, were marked with elevated H3K27ac modifications and possessed increased genome accessibility levels in *Kmt2d*‐deficient AML cells, concordant with increased gene expression and enhanced rRNA concentration (Figure S4D, Supporting Information). Meanwhile, we analyzed the KMT2D binding, H3K4me1, or H3K4me2 modification levels in Pol I & III binding sites and found that the KMT2D binding levels were dramatically reduced in rDNA regions compared to those in the whole genome. Similar results were obtained for the H3K4me1 or H3K4me2 modification levels (Figure S4E, Supporting Information). Further, we found that there was little difference in the KMT2D‐binding, H3K4me1, or H3K4me2 levels between *Kmt2d* knockdown and restored AML cells in Pol I or III binding sites (Figure S4F, Supporting Information). These data suggest that the role of *KMT2D* in rDNA expression is not likely through a direct effect on rDNA‐associated chromatin.

By comparing the KMT2D binding and histone modification levels between *Kmt2d* restored and knockdown AML cells, we found that 2516 peaks had a significant decrease of KMT2D binding levels and contained reduced H3K4me1, H3K4me2, and H3K27ac modifications in *Kmt2d*‐deficient AML cells, which indicated these regions were specifically bound by KMT2D (*p* < 0.05, log2‐fold change < −1; Figure S4G and Table S4, Supporting Information). These KMT2D‐specific binding sites were preferentially distributed in distal intergenic (38.92%), intron (38.31%), and promoter regions (13.20%), similar to previously reported^[^ ^15^, ^16^, ^21^, ^39^, ^40^ ^]^ (Figure S4H, Supporting Information). KMT2D‐targeted genes significantly overlapped with genes that had decreased H3K4me1 and H3K4me2 modification levels in sh*Kmt2d* AML cells. Specifically, 730 genes were identified with significant KMT2D, H3K4me1, and H3K4me2 downregulation (Figure S4I, Supporting Information). These genes also possessed significant decreases in H3K27ac modification, chromatin accessibility, and gene expression levels (Figure S4J, Supporting Information). Among them, 178 genes had significantly reduced expression levels and were considered as *KMT2D* directly regulated genes, in which we found one encoding a negative regulator of mTORC1, *Ddit4* ^[^ ^41^ ^]^ (Figure S4I, Supporting Information). KMT2D bound the near transcriptional start site (TSS) and the region around 20 kb upstream of the TSS of *Ddit4*, and caused a significant reduction in H3K4me1 and H3K4me2 occupations as well as gene expression (Figure 4E). The reduction of *Ddit4* expression was confirmed by qRT‐PCR (Figure 4F). To prove the role of *Ddit4* participated in the *Kmt2d*‐deficiency‐mediated hyperactivation of the mTOR pathway, we conduct a rescue experiment by overexpressing *Ddit4* in *Kmt2d*‐deficient AML cells (Figure S4K, Supporting Information). As a consequence, overexpressed *Ddit4* rescued almost all of the phenotypes associated with *Kmt2d* deficiency. Specifically, mTOR activation was inhibited evidenced by the impediment to the increase of phosphorylated ribosomal protein S6 (Figure 4G). *Ddit4* overexpression reduced the fluorescence intensity of nucleolar protein fibrillarin, rRNA transcriptions, and protein synthesis rate (Figure 4H–J). Eventually, we observed a significant inhibition of cell proliferation and a reduction of cell and nuclear sizes by *Ddit4* overexpression (Figure 4K and Figure S4L, Supporting Information). Hence, *Kmt2d* might directly control the expression of mTOR negative regulator *Ddit4*, whose deficiency activates the mTOR pathway and thus induces ribosome biogenesis.

### 2.5 *Kmt2d‐*Deficient AML Cells Are Sensitive to the Inhibitor of Ribosome Biogenesis

**Figure 5:**
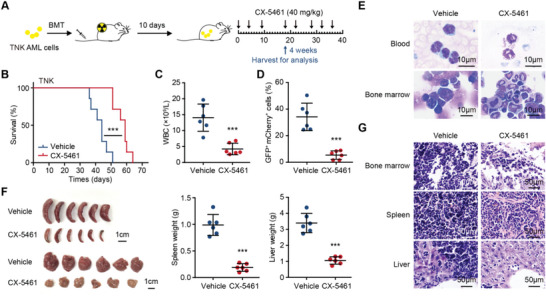
*Kmt2d‐*deficient AML is sensitive to the ribosome biogenesis inhibitor. A) Schematic experimental design. TNK (*Trp53* ^−/−^; sh*Nf1*; sh*Kmt2d*) AML cells were transplanted into sub‐lethally irradiated recipient mice. CX‐5461 or vehicle (0.5% CMC‐Na) was administered orally (40 mg kg^−1^) to recipient mice 10 days after transplant. The time points of drug administration are indicated. Mice were followed for AML development or sacrificed to analyze 4 weeks after transplant. B) Kaplan–Meier survival curves of leukemia mice treated with vehicle (blue; *n* = 7) or CX‐5461 (red; *n* = 7). ***p < 0.001 (log‐rank test). The results were the combination of two independent trials. C) WBC counts in the peripheral blood of CX‐5461 and vehicle‐treated leukemia mice 4 weeks after transplant (*n* = 6 per group). D) The percentage of GFP and mCherry double‐positive population in the peripheral blood of CX‐5461 and vehicle‐treated leukemia mice 4 weeks after transplant (*n* = 6 per group). E) Representative blood smears and bone marrow cytospins of CX‐5461 and vehicle‐treated leukemia mice 4 weeks after transplant. F) Representative images of spleens and livers (left) and quantitation of their weights (right) in CX‐5461 and vehicle‐treated leukemia mice 4 weeks after transplant (*n* = 6 per group). G) Representative images of histological analyses of bone marrow, spleen, and liver of CX‐5461 and vehicle‐treated leukemia mice 4 weeks after transplant. C,D,F) Graph represents the mean ± SD, ***p < 0.001 (unpaired two‐tailed *t*‐test).

To investigate the impact of ribosome biogenesis on *Kmt2d*‐deficient leukemia cell growth, we treated mice bearing sh*Kmt2d* AML with CX‐5461, an inhibitor of rRNA synthesis (**Figure** **5**A). CX‐5461 could directly and selectively target Pol I‐mediated transcription by disrupting the binding of the SL1 transcription factor to the promoter of ribosomal RNA genes.^[^ ^42^ ^]^ CX‐5461 treatment significantly prolonged the survival of recipient mice transplanted with sh*Kmt2d* AML cells compared to the vehicle‐treated group (Figure 5B). We repeated this experiment and harvested recipient mice simultaneously 4 weeks after the transplant. We found that sh*Kmt2d* leukemia cells in the peripheral blood of CX‐5461‐treated mice were significantly reduced compared to those in control vehicle‐treated mice, as shown by decreased WBC counts and the percentages of GFP and mCherry double‐positive population (Figure 5C,D). Blast cells were also dramatically reduced as shown in the blood smears and bone marrow cytospins (Figure 5E). The harvested spleens and livers of CX‐5461‐treated mice had reduced size, weight, and a diminished infiltration of leukemia cells as examined by H&E staining, compared to splenomegaly and hepatomegaly correlated with severe infiltration of leukemia cells in the vehicle‐treated group (Figure 5F,G). We also compared the response to CX‐5461 in *Kmt2d*‐deficient leukemia cells (TNK) and normal *Kmt2d* control leukemia cells (TN) side by side. Results showed that *Kmt2d*‐deficient AML cells were more sensitive to the CX‐5461 treatment (Figure S5A, Supporting Information). Taken together, our data showed that CX‐5461 is an effective drug for treating *Kmt2d*‐deficient AML, indicating a ribosome biogenesis vulnerability.

### 2.6 *KMT2D* Deficiency Upregulates Ribosome Biogenesis in Human AML

**Figure 6:**
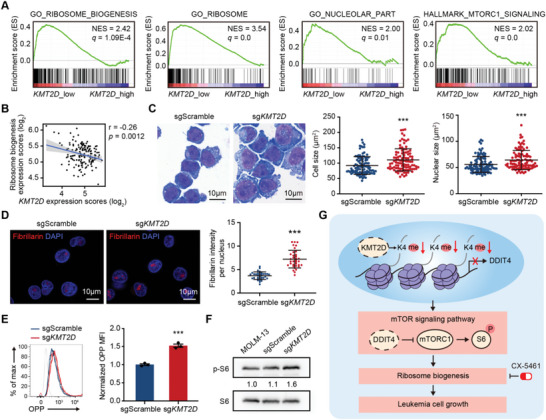
*KMT2D* regulates ribosome biogenesis in human AML. A) GSEA showing the positive enrichment of the GO_RIBOSOME_BIOGENESIS, GO_RIBOSOME, GO_NUCLEOLAR_PART, and HALLMARK_MTORC1_SIGNALING gene sets in *KMT2D* low expression AML patients (*n* = 20) compared to *KMT2D* high expression ones (*n* = 122) in the TCGA‐LAML cohort. B) Scatter plot showing the negative correlation between expression levels of *KMT2D* and ribosome biogenesis‐related genes in the TCGA‐LAML cohort. *p* Values were determined by the two‐tailed Student's *t*‐test. *r*, Pearson's correlation coefficient. C) Representative pictures performed on Liu's‐stained cytospins (left) and quantitation of cell size and nuclear size (right) of sgScramble and sg*KMT2D* MOLM‐13 cell lines (*n* = 100 per group). D) Representative immunofluorescence images for nucleolar protein Fibrillarin (left) and quantitation of fluorescence intensity per nucleus (right) of sgScramble and sg*KMT2D* MOLM‐13 cell lines (*n* = 35 per group). E) Representative flow cytometric profile (left) and quantitation of OPP MFI in sgScramble and sg*KMT2D* MOLM‐13 cell lines (*n* = 3 per group). F) Western blotting showing the phosphorylation levels of ribosomal protein S6 in sgScramble and sg*KMT2D* MOLM‐13 cell lines. G) Schematic diagram showing the working model of *KMT2D* deficiency in leukemia cell growth. C,D,F) Quantitation was analyzed by ImageJ. C,D,E) Graph represents the mean ± SD, ***p < 0.001 (unpaired two‐tailed *t*‐test).

To translate our findings into human settings, we analyzed the transcriptomic profiles of 142 AML patients in the TCGA‐LAML cohort.^[^ ^4^ ^]^ GSEA results showed that upregulated genes in *KMT2D* low expression AML patients were significantly positively enriched in GO_ribosome_biogenesis, GO_ribosome, GO_nucleolar_part, GO_rRNA_metabolic_process, HALLMARK_mTORC1_signaling, GO_translational_initiation, and GO_translational_elongation pathways, compared to *KMT2D* high expression ones (**Figure** **6**A and Figure S6A, Supporting Information). Consistently, *KMT2D* expression was negatively correlated with the expressions of ribosomal biogenesis‐related genes (Figure 6B).

Additionally, we also investigated the correlation between *KMT2D* and ribosome biogenesis in human AML cell line MOLM‐13. We first constructed *KMT2D*‐deficient MOLM‐13 cells with CRISPR/Cas9 genome editing technology (Figure S6B,C, Supporting Information). Consistent with our previous findings, upregulated ribosome biogenesis was observed in *KMT2D*‐mutated MOLM‐13 cells. Compared to control cells (sgScramble), we found that in *KMT2D*‐mutated cells, cell and nuclear sizes were enlarged (Figure 6C); fibrillarin staining was significantly increased (Figure 6D); rRNA concentration was enhanced (Figure S6D, Supporting Information). More importantly, translation activity in *KMT2D*‐mutated MOLM‐13 cells was enhanced as measured by OPP incorporation (Figure 6E). Furthermore, we observed that the mTOR pathway was activated in sg*KMT2D* cells, as shown by increased phosphorylated S6 levels (Figure 6F), and rapamycin treatment reduced the cell and nuclear size (Figure S6E, Supporting Information). Altogether, these results supported that our findings of *KMT2D*‐deficiency‐inducing ribosome biogenesis are conserved in human AML samples.

In summary, our study identified *KMT2D* as a tumor suppressor gene, whose deficiency was critical for AML tumorigenesis. *KMT2D* deficiency reduced H3K4 methylation levels and suppressed the expression of *DDIT4*, the negative regulator in the mTOR signaling pathway, which led to the activation of the mTOR pathway and enhancement of ribosome biogenesis, thus contributing to AML development (Figure 6G).

## 3 Discussion

*KMT2D* is one of the most frequently mutated genes in human cancers. Its loss‐of‐function mutations or loss have been demonstrated as driving forces for the initiation and development of many cancers like B‐cell lymphoma, melanoma, medulloblastoma, lung, and pancreas cancers.^[^ ^15^, ^16^, ^17^, ^18^, ^19^, ^20^, ^21^ ^]^ In spite of the well‐established tumor suppressive effect of *KMT2D* in lymphoma and solid cancers as well as *KMT2C* and *KDM6A* encoding components in the COMPASS‐like complex were identified as tumor suppressor genes in AML, the role of *KMT2D* in myeloid malignancies seems the opposite. Nussenzweig and Hess's groups found that *Kmt2d* deficiency prevents the *MLL‐AF9*‐ or *HOXA9*‐driven AML formation in mice.^[^ ^26^, ^27^ ^]^ They further illustrated that *Kmt2d* deficiency induced myeloid differentiation in *MLL‐AF9* leukemic blasts, suggesting a tumor‐promoting role of *Kmt2d*.^[^ ^26^ ^]^ However, we found that *KMT2D* expressions were generally lower in AML cells compared to normal peripheral blood cells, suggesting that *KMT2D* may have a tumor‐suppressive function in some AMLs. Functional studies demonstrated that *Kmt2d* deficiency together with *Trp53* and *Nf1* loss in hematopoietic stem and progenitor cells could promote AML in recipient mice, supporting that *KMT2D* is a tumor suppressor in *TP53* ^−/−^ AML. One gene, having a distinct or even opposite impact on AML with different genetic alterations or in different phases, has been reported before. *SETD2* loss drove leukemogenesis when cooperated with NUP93‐HOXD13,^[^ ^43^ ^]^ while inhibiting *MLL‐AF9* AML cell growth both in vitro and in vivo.^[^ ^44^ ^]^ *Ezh2* acts as a tumor suppressor during AML induction, while it exerts an oncogenic function during disease maintenance.^[^ ^45^ ^]^

As an important tumor suppressor gene, *KMT2D* regulates the expressions of genes involved in multiple cellular functions like differentiation and metabolism reprogramming in cancer cells.^[^ ^17^, ^19^, ^26^ ^]^ Here, we revealed a new role of *KMT2D*, enhancing ribosome biogenesis when repressed. We found that when *Kmt2d* was suppressed, the synthesis of ribosome RNA, the expressions of ribosome biogenesis‐related genes and genes encoding RNA polymerase I or III complexes were increased. Consequently, the nucleolus was enlarged and more newly synthesized peptides were produced in *Kmt2d*‐deficient cells. This correlation between *KMT2D* defect and enhanced ribosome biogenesis is consistent in human AML cell lines and AML patients. From the multiomics analysis, we observed that the hyperactivated mTOR pathway was associated with *KMT2D* deficiency, and mTORC1 inhibitor rapamycin could reduce ribosome biogenesis. The mTORC1 signaling pathway has been reported to control ribosome biogenesis in multiple steps, including ribosome protein translation and ribosome RNA transcription.^[^ ^38^, ^46^ ^]^ We identified that KMT2D could bind to and regulate the expression of *Ddit4*, encoding a negative regulator of mTORC1. The overexpression of *Ddit4* could repress *Kmt2d* deficiency‐induced upregulation of ribosome biogenesis, suggesting that *KMT2D* has an impact on translation likely through *DDIT4*‐mTOR. Meanwhile, we would not exclude other possible factors and pathways that may be involved. We noticed that genes in MYC_TARGETS were significantly positively enriched in *Kmt2d*‐deficient HSPCs and AML cells (unpublished data). Therefore, *KMT2D* may also regulate ribosome biogenesis and leukemogenesis through mTOR‐independent mechanisms. Altogether, we illustrated a novel function of *KMT2D* that could regulate protein translation in addition to its critical role in controlling gene transcription.

Furthermore, our work revealed that CX‐5461, a specific RNA polymerase I inhibitor, could effectively reduce tumor burden and significantly prolong the survival of mice bearing *Kmt2d*‐deficient AML. The drug experiment not only confirmed the important role of ribosome biogenesis in *Kmt2d*‐deficiency‐induced AML, but also suggested a potential therapeutic target for *KMT2D* low‐expression or mutated AML patients. Ribosome biogenesis has emerged as an effective pathway for cancer therapeutics. Apart from chemotherapeutic drugs which exert their cytotoxic effects by perturbation of ribosome biogenesis at various levels, several novel compounds that selectively target ribosome production or function, mainly inhibitors of Pol I transcription, such as CX‐5461, have recently entered clinical trials.^[^ ^47^, ^48^, ^49^ ^]^ CX‐5461 has shown therapeutic potential in hematological malignancies. CX‐5461 treatment could effectively inhibit mouse *MLL*‐fusion AML progression and human AML cell lines in both *Trp53*‐dependent and ‐independent mechanisms.^[^ ^50^, ^51^ ^]^ Also, combination therapy targeting ribosome biogenesis and mTORC1‐dependent translation synergistically extends survival in *MYC*‐driven lymphoma.^[^ ^52^ ^]^ Thus, several phase I clinical trials of CX‐5461 in B‐cell lymphomas and solid cancers have been done^[^ ^53^ ^]^ or are ongoing (NCT02719977, NCT04890613, and NCT05425862). According to these studies, CX‐5461 treatment in patients is safe and holds promise.^[^ ^53^ ^]^ Further validation of CX‐5461 and other inhibitors with similar functions in human cancer cells with *KMT2D* mutations or deficiency would pave the way for their potential applications.

In summary, we demonstrate that *KMT2D* could be a tumor suppressor gene in AML. *KMT2D* regulates the expression of a negative regulator *DDIT4* in the mTOR pathway via histone methyltransferase activity, suppresses ribosome biogenesis, and eventually prevents leukemogenesis. CX‐5461, the selective inhibitor of RNA Pol I transcription, has an antileukemia effect on *Kmt2d*‐deficient AML. Besides, *KMT2D* mutations and abnormalities are also common in other leukemias.^[^ ^54^, ^55^ ^]^ As such, our observations may have implications beyond AML.

## 4 Experimental Section

### Mice

*Trp53* ^−/−^ mice from The Jackson Laboratory (Stock No: 002101, RRID:IMSR_JAX:002101)^[^ ^56^ ^]^ and *Trp53* ^−/−^ Cas9‐expressing mice obtained by breeding *Trp53* ^−/−^ mice with *Cas9* knock‐in mice from The Jackson Laboratory (Stock No: 026179, RRID:IMSR_JAX:026179)^[^ ^57^ ^]^ were served as donor mice (both in C57BL/6 background). Bone marrow cells were freshly isolated from femurs and tibias of 6 to 8 weeks old donor mice and HSPCs were sorted using c‐kit (CD117) Microbeads (Miltenyi Biotec Cat# 130‐091‐224, RRID:AB_2753213) and a QuadroMACS separator (Biocompare, South San Francisco, CA). For in vivo tumorigenesis, HSPCs were transduced with lentiviruses encoding sgRNAs or retroviruses encoding shRNAs, and 10E+6 transfected HSPCs were injected into 5.5 Gy irradiated C57BL/6 recipient mice (Cat# 219, Charles River) via the tail vein injection. All recipient mice were randomly divided into each group before transplantation and monitored by complete blood count (CBC) and blood smears. Blood count measurement was performed on a HemaVet 950FS Blood Analyzer (RRID:SCR_020016). For secondary transplant experiments, 10E+6 bone marrow leukemia cells were injected into 5.5 Gy irradiated C57BL/6 recipient mice.

### Cell Culture

HEK293T (RRID:CVCL_0063) and NIH 3T3 (ATCC Cat# CRL‐1658, RRID:CVCL_0594) were from ATCC and cultured at 37 °C with 5% CO_2_ in Dulbecco's modified Eagle medium (DMEM) supplemented with 10% vol/vol fetal bovine serum (FBS) and penicillin (100 U mL^−1^)/streptomycin (0.1 mg mL^−1^). MOLM‐13 (RRID:CVCL_2119) was from DSMZ and cultured at 37 °C with 5% CO_2_ in RPMI 1640 medium supplemented with 10% FBS and penicillin (100 U mL^−1^)/streptomycin (0.1 mg mL^−1^). Mouse HSPCs and AML cells were cultured at 37 °C with 7.5% CO_2_ in BCM medium [45% IMDM + 45% DMEM + 10% FBS + 50 × 10^−6^ m *β*‐mercaptoethanol + penicillin (100 U mL^−1^)/streptomycin (0.1 mg mL^−1^)] supplemented with 10% stem cell medium (SCM) including mIL‐3 (10 ng mL^−1^, Cat# 403‐ML; R&D Systems), mIL‐6 (10 ng mL^−1^, Cat# 406‐ML; R&D Systems), and mSCF (50 ng mL^−1^, Cat# 455‐MC; R&D Systems).

### shRNA Construction

97 bp oligonucleotides with gene‐specific hairpins were designed by splashRNA (http://splashrna.mskcc.org/). Sequences of shRNAs for *Kmt2d* are displayed in Table S5 in the Supporting Information. Sequences of sh*Ren* and sh*Nf1* are from published reports.^[^ ^8^ ^]^ shRNAs were cloned into retroviral vectors including MSCV‐shRNA‐SV40‐GFP, MSCV‐shRNA‐SV40‐mCherry, MSCV‐shRNA‐PGK‐puromycin‐IRES‐GFP, MSCV‐shRNA‐IRES‐rtTA3, and TRE‐dsRed‐shRNA‐PGK‐Venus‐IRES‐neomycin.

### Genome Editing

sgRNAs were designed by the ATUM CRISPR gRNA Design tool (https://www.atum.bio/eCommerce/cas9/input). Sequences of sgRNAs for *Kmt2d*, *Nf1*, *Cas9*, Scramble, and *KMT2D* are displayed in Table S6 in the Supporting Information. sgRNAs were cloned into lentiviral vector U6‐sgRNA‐U6‐sgRNA‐U6‐sgRNA‐EFS‐NS‐mCherry and U6‐sgRNA‐EFS‐NS‐mCherry. To construct *KMT2D*‐mutated MOLM‐13 cell lines, V2T plasmid which expressed *Cas9* cDNA and puromycin was transduced into cells, followed by puromycin (1 µg mL^−1^, Cat# A1113803, Thermo Fisher Scientific, Waltham, MA) selection, and sgRNA transduction. Mutation validation was performed by the T7E1 (Cat# EN303‐01, Vazyme, Nanjing, China) assay and primers for the T7 endonuclease I mismatch detection assay are displayed in Table S7 in the Supporting Information.

### cDNA Cloning

*Ddit4* cDNA sequences were obtained from the cDNA library of mouse AML cells and cloned into retroviral vector MSCV‐cDNA‐IRES‐GFP.

### Flow Cytometry

Flow cytometry analyses were performed on the BD LSRFortessa Flow Cytometer (BD Biosciences, San Jose, CA). Data were analyzed using FlowJo software (RRID:SCR_008520).^[^ ^58^ ^]^ Antibodies used in flow cytometry are displayed in Table S8 in the Supporting Information.

### Western Blotting

10E+6 cells were harvested and lysed in sodium dodecyl sulfate (SDS) buffer (50 × 10^−3^ m Tris‐HCl (pH 6.8), 2% w/v SDS, 150 × 10^−3^ m NaCl, 1% NP‐40, 40 × 10^−3^ m dithiothreitol) followed by sonication with an ultrasonic cell disruptor. Lysate proteins were separated by 12% SDS‐polyacrylamide gel electrophoresis gels and transferred to polyvinylidene fluoride membranes. The primary antibodies used are displayed in Table S9 in the Supporting Information. Relative protein gray‐scale values were analyzed using ImageJ (RRID:SCR_003070).^[^ ^59^ ^]^

### Quantitative Real‐Time PCR

Total RNA was extracted with TRIzol Reagent (Cat# 15596026, Thermo Fisher Scientific) and cDNA was synthesized using Hiscript III RT SuperMix for qPCR (Cat# R323‐01, Vazyme) following the manufacturer's instructions. qRT‐PCR was performed using ChamQ Universal SYBR qPCR Master Mix (Cat# Q711‐02, Vazyme) on the QuantStudio 3 Real Time PCR System (RRID:SCR_018712). Sequences of qRT‐PCR primers are displayed in Table S10 in the Supporting Information.

### H&E and Immunofluorescence Staining

For H&E staining, mouse organs were fixed overnight in 4% paraformaldehyde, embedded in paraffin blocks, and sectioned. Tissue sections were stained with hematoxylin (Cat# 03971, Sigma‐Aldrich, Shanghai, China) for 5 min to identify the cell nucleus and eosin (Cat# 318906, Sigma‐Aldrich) for 5 min to identify the cytoplasm. For immunofluorescence, cells were deposited on slides by cytocentrifuge (CytoSpin 4, Thermo Fisher Scientific), fixed in 4% paraformaldehyde for 15 min and permeabilized with 0.3% Triton X‐100 for 15 min. The antibody of Fibrillarin (Cell Signaling Technology Cat# 2639, RRID:AB_2278087) was used. After secondary antibodies were added, the slides were mounted by mounting medium with 4′,6‐diamidino‐2‐phenylindole (Cat# C0060, Solarbio Life Science, Beijing, China). And images were taken with Zeiss LSM 880 with Airyscan confocal laser scanning microscope (RRID:SCR_020925).

### Analysis of rRNA Synthesis by 5‐FUrd Incorporation

Cells were incubated for 30 min in medium containing 2 × 10^−3^ m 5‐FUrd (Cat# F5130, Sigma‐Aldrich), then washed in cold phosphate‐buffered saline for subsequent immunofluorescence, performed as described above, using the antibody of BrdU (Sigma‐Aldrich Cat# B2531, RRID:AB_476793).

### OPP Protein Synthesis Assay

Nascent protein synthesis was detected using Click‐iT Plus OPP Protein Synthesis Assays kit (Cat# C10458, Thermo Fisher Scientific) following the manufacturer's instructions. The intensity of OPP signals was detected by flow cytometry analyses and presented as the mean fluorescence index.

### In Vivo Treatment

10E+6 bone marrow leukemia cells were injected into 5.5 Gy irradiated C57BL/6 recipient mice. Drug treatments were initiated 10 days after transplantation. Mice were treated with either vehicle (0.5% CMC‐Na) or 40 mg kg^−1^ CX‐5461 (Cat# S2684, Selleck, Shanghai, China) every 3 days via oral gavage. Administration timing can be adjusted according to the physical condition of the mice. The leukemia progression in recipient mice was monitored by CBC, flow cytometry, and blood smears.

### RNA‐seq Analysis

AML cells treated with doxycycline (sh*Kmt2d*) or without doxycycline (*Kmt2d*‐restored) were harvested for RNA‐seq. HSPCs isolated from bone marrow cells of *Trp53* ^−/−^ mice were transfected with retroviruses encoding sh*Kmt2d* or sh*Ren*. 48 h later GFP‐positive cells were sorted by the BD FACSAria III cell sorter (RRID:SCR_016695) for RNA‐seq. RNA libraries were prepared using standard Illumina protocols. Transcriptome sequencing was performed by the Illumina NovaSeq 6000 platform. RNA‐seq reads were aligned to the reference mouse genome (mm10) by STAR (v 2.6.0, RRID:SCR_004463).^[^ ^60^ ^]^ Raw counts normalization, significance scores, and log2‐fold change values calculation were performed by DESeq2 (RRID:SCR_015687).^[^ ^61^ ^]^ Differential expression genes were used for GO analysis with R package clusterProfiler (RRID:SCR_016884).^[^ ^62^ ^]^ GSEA (RRID:SCR_003199) was performed to identify significantly enriched pathways. The software Integrative Genomics Viewer (IGV, RRID:SCR_011793) and the R packages ggpubr (RRID:SCR_021139), ggplot2 (RRID:SCR_014601), pheatmap (RRID:SCR_016418), Vennerable were used for data visualization.

### CUT&Tag Analysis

sh*Kmt2d* and *Kmt2d*‐restored AML cells were harvested for CUT&Tag assay using NovoNGS CUT&Tag 3.0 High‐Sensitivity Kit (Cat# N259‐YH01‐01A, NovoProtein, Suzhou, China). The primary antibodies used are displayed in Table S9 in the Supporting Information. Genomic sequencing was performed on the Illumina NovaSeq 6000 platform. After quality control by fastp (RRID:SCR_016962)^[^ ^63^ ^]^ standard workflow, Bowtie2 (RRID:SCR_016368) was used for mouse genome (mm10) index construction and 150 bp paired‐end clean data alignments with “–local –very‐sensitive‐local –no‐unal –no‐mixed –no‐discordant –phred33 ‐I 10 ‐X 700” option.^[^ ^64^ ^]^ The program SAMtools (RRID:SCR_002105) was performed to convert sam files into sorted bam files. Duplicates were removed using MarkDuplicates tools from Picard (RRID:SCR_006525) with “REMOVE_DUPLICATES = true” option. Normalized bw files were generated by DeepTools (RRID:SCR_016366) with “bamCoverage ‐bs = 1 –normalizeUsing BPM” option for further IGV (RRID:SCR_011793) visualization. MACS2 was performed for peak calling with “macs2 callpeak –broad ‐q 1e‐2 ‐f BAMPE ‐g mm –keep‐dup all” option.^[^ ^65^ ^]^ Genomic distribution of peaks was identified by ChIPseeker (RRID:SCR_021322) with “annotatePeak” option and the TSS region “tssRegion” was set as (−3000, 3000).^[^ ^66^ ^]^ “narrowPeak” files were transformed into GrangesList forms and peaks were converted into consensus counts. Data normalization and difference comparison were performed using DESeq2 (RRID:SCR_015687). Peaks with a *p*‐value cutoff of 0.05 (Wald test) were identified as differential binding or modification regions for downstream analyses.

### ATAC‐seq Analysis

Library preparation was performed as previously described,^[^ ^67^ ^]^ and the transposase was from TruePrep DNA Library Prep Kit V2 for Illumina (Cat# TD501, Vazyme, Nanjing, China). The library was sequenced on the Illumina NovaSeq 6000 platform. NGmerge was performed to remove adapters from 150 bp paired‐end raw data with “NGmerge ‐a ‐v ‐n 20” option.^[^ ^68^ ^]^ The mouse genome (mm10) index construction and follow‐up alignments were performed using Bowtie2 (RRID:SCR_016368) with “–very‐sensitive ‐X2000 ‐x mm10” option. Duplicates were removed using MarkDuplicates tools from Picard (RRID:SCR_006525). The mitochondrial genome contamination was removed and bam files were obtained using SAMtools (RRID:SCR_002105) and awk commands. Normalized bw files were generated using DeepTools (RRID:SCR_016366) with “bamCoverage ‐bs = 1 –normalizeUsing BPM” option for further IGV (RRID:SCR_011793) visualization. The standard workflow of HMMRATAC^[^ ^69^ ^]^ was utilized for the ATAC‐seq peak calling step. The genomic distribution of accessibility sites was identified by ChIPseeker (RRID:SCR_021322) with “annotatePeak” option and the TSS region “tssRegion” was set as (−3000, 3000). “gappedPeak” files were transformed into GrangesList forms and peaks were converted into consensus counts. Data normalization and difference comparison were performed using DESeq2 (RRID:SCR_015687). Pol I and Pol III binding regions were cited from GSE145874.^[^ ^70^ ^]^ Peaks with a *p*‐value cutoff of 0.05 (Wald test) were identified as differential accessibility regions for downstream analyses.

### Referred AML Patient Transcriptome Data Analysis

Genetic alteration data were analyzed from The Cancer Genome Atlas AML project (TCGA‐LAML)^[^ ^4^ ^]^ and OHSU AML cohorts.^[^ ^30^ ^]^ The co‐occurrence of *KMT2D*, *TP53*, and *NF1* mutations in leukemia patients was analyzed by GENIE Cohort v11.0‐public datasets (https://genie.cbioportal.org/, *n* = 4670). Transcriptome data of normal and AML patients were acquired from high‐throughput sequencing data GSE48173 (AML, *n* = 43; normal, *n* = 17)^[^ ^28^ ^]^ and microarray data GSE1159 (AML, *n* = 285; normal, *n* = 8).^[^ ^29^ ^]^ RPKM of GSE48173 was performed with log2 transformation to obtain the standardized expression value. *p* value was determined by the Wilcoxon signed‐rank test. To estimate the impact of *KMT2D* expression on the prognosis of AML patients, patients were divided into *KMT2D*‐high and *KMT2D*‐low groups by FPKM in the TCGA‐LAML^[^ ^4^ ^]^ and Beat AML cohorts.^[^ ^30^ ^]^ The optimal cut‐point for numerical variables of *KMT2D* expression was calculated with the maximally selected rank statistics by the survminer (RRID:SCR_021094) package to stratify patients for the survival analysis, set as 20.78 and 7.12 in TCGA‐LAML and Beat AML cohort, respectively. According to the algorithm, 122 and 20 patients in the TCGA‐LAML cohort were divided into *KMT2D*‐high and *KMT2D*‐low groups. GSEA (RRID:SCR_003199) was performed to identify significantly enriched pathways between the two groups. The ribosome biogenesis gene signature was determined by GOBP_RIBOSOME_BIOGENESIS.^[^ ^71^ ^]^ Correlations between *KMT2D* expression and mean expression levels of genes involved in ribosome biogenesis were visualized by ggpubr (RRID:SCR_021139) and correlation values were determined by Spearman's rank correlation coefficient.

### Data Visualization

R packages ggpubr and ggplot2 were performed to show the expression and accessibility level differences by box plots. Heatmaps were generated by the R package pheatmap to present differentially expressed genes. Bar plots and dot plots of GO and GSEA enrichment results were drawn by ggplot2. The program DeepTools was utilized to visualize the histone modification levels. Matrix computations of differential modification and accessibility peaks were performed using “ComputeMatrix reference‐point –referencePoint center ‐b 3000 ‐a 3000 –skipZeros” option.

### Statistical Analysis

Statistical test methods, sample sizes, and *p* values are indicated in the corresponding figure legends. Statistical significance was determined using GraphPad Prism (v5.01, RRID:SCR_002798) and PASW Statistics18 software by unpaired two‐tailed *t*‐test, Wilcoxon signed‐rank test, Wald test, hypergeometric distribution, or log‐rank test. Statistical significance was defined as *p* < 0.05. Error bars were shown as SD.

### Study Approval

All animal study procedures and experiments were reviewed and approved by the Experimental Animal Ethics Committee of the State Key Laboratory of Biotherapy and Cancer Center, Sichuan University (approval number: 20170209) and were in accordance with the 8th edition of the Guide for the Care and Use of Laboratory Animals.

## Conflict of Interest

The authors declare no conflict of interest.

## Author Contributions

J.X., A.Z., and S.Z. contributed equally to this work. C.C. and Y.L. conceived the project and designed experiments. J.X., S.Z., M.C., L.Z., X.H., J.Z., B.W., X.D., Z.W., L.Q., K.S., S.L., Y.W., Q.Z., and P.L. performed experiments. J.X., A.Z., S.Z., R.P.G., and T.N. analyzed data. J.X., A.Z., X.C., and X.P. performed RNA‐seq, ATAC‐seq, and CUT&Tag analyses. J.X., A.Z., X.C., M.W., C.C., Y.L., and T.N. prepared the manuscript. J.X., A.Z., and S.Z. are co‐first authors. J.X. and S.Z. performed much of the experimental work, and A.Z. performed much of the bioinformatic analyses. All authors read and approved the final manuscript.

## Supporting information

Supporting Information

## Data Availability

The data that support the findings of this study are openly available in GEO at https://www.ncbi.nlm.nih.gov/geo/query/acc.cgi?acc=GSE147210, reference number 147210.
