# Supplementary material for: KMT2D Deficiency Promotes Myeloid Leukemias which Is Vulnerable to Ribosome Biogenesis Inhibition
Source: Adv Sci (Weinh). 2023 May 4;10(19):2206098. doi: 10.1002/advs.202206098 (PMC10323629; doi:10.1002/advs.202206098)
Supplement: Supplementary file 1 — Supporting Information [file ADVS-10-2206098-s001.pdf]

ADVANCED SCIENCE
Open Access

Supporting Information

for *Adv. Sci.*, DOI 10.1002/advs.202206098

KMT2D Deficiency Promotes Myeloid Leukemias which Is Vulnerable to Ribosome Biogenesis Inhibition

Jing Xu, Ailing Zhong, Shan Zhang, Mei Chen, Lanxin Zhang, Xiaohang Hang, Jianan Zheng, Baohong Wu, Xintong Deng, Xiangyu Pan, Zhongwang Wang, Lu Qi, Kaidou Shi, Shujun Li, Yiyun Wang, Manli Wang, Xuelan Chen, Qi Zhang, Pengpeng Liu, Robert Peter Gale, Chong Chen, Yu Liu and Ting Niu**

SUPPLEMENTARY INFORMATION

This file includes:

Supplementary Tables

Supplementary Tables 1-4 and Table 10 were provided as Excel files.

Supplementary Tables 5-9 were provided in this document.

Supplementary Figures

Supplementary Figure 1

Supplementary Figure 2

Supplementary Figure 3

Supplementary Figure 4

Supplementary Figure 5

Supplementary Figure 6

Supplementary Tables

Supplementary Table 1. *KMT2D* alterations, expression profiling and relevant prognosis in AML patients, related to Figure 1, Supplementary Figure 1 and 2.

Supplementary Table 2. Gene expression profiling of *Kmt2d* knockdown versus *Kmt2d* restored AML cells, related to Figure 3 and Supplementary Figure 3.

Supplementary Table 3. Gene expression profiling of sh*Kmt2d* versus sh*Ren* HSPCs, related to Supplementary Figure 3.

Supplementary Table 4. Differential KMT2D binding regions, H3K4me1, H3K4me2 and H3K27ac modification regions and accessibility regions in *Kmt2d* knockdown and *Kmt2d* restored AML cells, related to Figure 4 and Supplementary Figure 4.

Supplementary Table 5. Sequences of shRNA.

shRNA	Sequence
sh*Kmt2d*-1	5'-TGCTGTTGACAGTGAGCGCCGGCCTGAGTTTGTGATCAAATAGTGAAG CCACAGATGTATTTGATCACAAACTCAGGCCGTTGCCTACTGCCTCGGA-3'
sh*Kmt2d*-2	5'-TGCTGTTGACAGTGAGCGAGCAGTGGATGTCTCAGATGAATAGTGAAG CCACAGATGTATTCATCTGAGACATCCACTGCCTGCCTACTGCCTCGGA-3'

Supplementary Table 6. Sequences of sgRNA.

sgRNA	Sequence
sg*Kmt2d* #1	Forward: 5'-GCTGAAGGGCTGGCGTTGTG-3'
	Reverse: 5'-CACAACGCCAGCCCTTCAGC-3'
sg*Kmt2d* #2	Forward: 5'-GCCGTTCCTTGCCGCTCACC-3'
	Reverse: 5'-GGTGAGCGGCAAGGAACGGC-3'
sg*Nf1*	Forward: 5'-GCTGCAGTGGAAGACCAGCG-3'
	Reverse: 5'-CGCTGGTCTTCCACTGCAGC-3'
sg*Cas9*	Forward: 5'-GGGCGTACTGGTCGCCGATC-3'
	Reverse: 5'-GATCGGCGACCAGTACGCCC-3'
sgScramble	Forward: 5'-GACATTTCTTTCCCCACTGG-3'

	Reverse: 5’-CCAGTGGGGAAAGAAATGTC-3’
sg*KMT2D*	Forward: 5’-GAGCCAGAAGCTGGCTGGTG-3’
	Reverse: 5’-CACCAGCCAGCTTCTGGCTC-3’

Supplementary Table 7. Primers for T7 endonuclease I assay.

sgRNA	Primer sequence
sg*Kmt2d* #1 T7-PCR-Forward	5’-GACACTGGATTAGCAGGTCTTC-3’
sg*Kmt2d* #1 T7-PCR-Reverse	5’-CAGGCGGATCTTTGTGAGTT-3’
sg*Kmt2d* #2 T7-PCR-Forward	5’-TCCAGACAGGCTCCATGATA-3’
sg*Kmt2d* #2 T7-PCR-Reverse	5’-GCGTTGTGCTCTCTGTAACT-3’
sg*KMT2D* T7-PCR-Forward	5’-TCCTCATGTGCCCTCAAAC-3’
sg*KMT2D* T7-PCR-Reverse	5’-ATGGTGATGTCCTCGTAGAAAC-3’

Supplementary Table 8. Antibodies used in flow cytometry.

Antigen-fluorochrome	Manufacturer	Clone	Cat#
CD11b-PB	BioLegend	M1/70	101224
Gr-1-PB	BioLegend	RB6-8C5	108430
B220-APC	BioLegend	RA3-6B2	103212
CD3ε-APC	BioLegend	145-2C11	100312
c-Kit-PE/Cy7	BioLegend	2B8	105814

Supplementary Table 9. Antibodies used in western blotting and CUT&Tag analysis.

Antigen	Manufacturer	Cat#	RRID
H3	HUABIO	EM30605	RRID:AB_2890027
H3K4me1	Abcam	ab8895	RRID:AB_306847
H3K4me2	Abcam	ab7766	RRID:AB_2560996
H3K27ac	Abcam	ab4729	RRID:AB_2118291
RPS6	abClonal	A0932	RRID:AB_2757466

p-S6	Cell Signaling Technology	2211S	RRID:AB_331679
KMT2D	MilliporeSigma	ABE1867	N/A

Supplementary Table 10. Primers for qRT-PCR.

Supplementary Figures

Supplementary Figure 1, related to Figure 1.

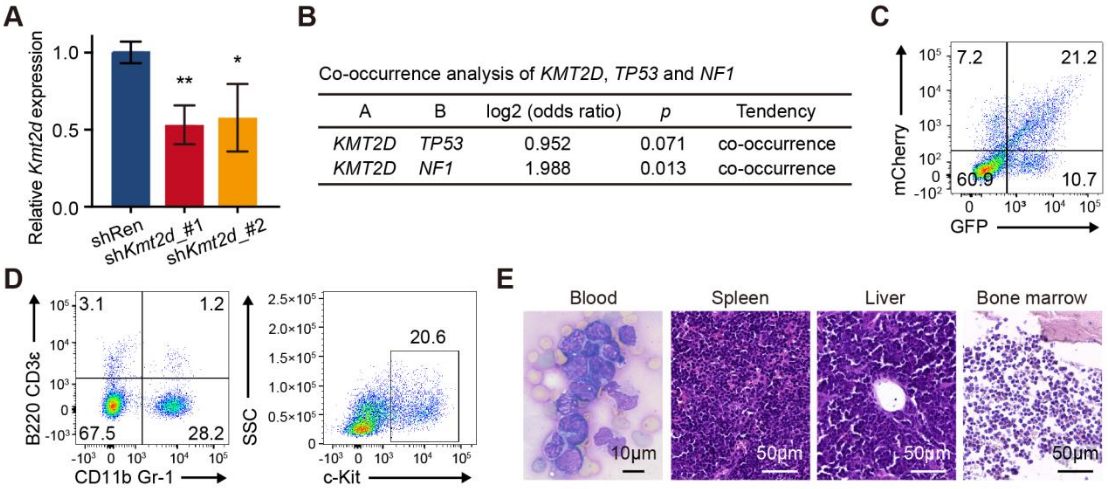

Supplementary Figure 1. *Kmt2d* knockdown or mutation promotes AML in mice.

(A) Relative mRNA levels of *Kmt2d* in mouse 3T3 cells were quantified by qRT-PCR (normalized to *Actin*; n = 3 *per* group). Graph represents the mean ± SD, *$p < 0.05$, **$p < 0.01$ (unpaired two-tailed *t*-test).

(B) Co-occurrence of *KMT2D*, *TP53* and *NF1* alterations in leukemia samples (n = 4670). Data were analyzed from GENIE Cohort v11.0-public datasets. *P* values were determined by Mutual Exclusivity Modules, provided by cBioPortal.

(C) Representative flow cytometric profiles showing the expression of GFP and mCherry in sh*Kmt2d*/sh*Ren* and sh*Nf1* transduced HSPCs pre-injection.

(D) Representative flow cytometric profiles showing the expression of CD11b/Gr-1, B220/CD3ε and c-Kit in bone marrow cells of secondary transplant recipient mice.

(E) Representative images of histological analyses of blood, spleen, liver and bone marrow of secondary transplant recipient mice.

Supplementary Figure 2, related to Figure 2.

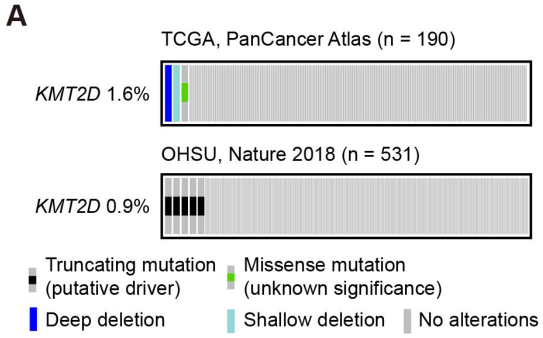

Supplementary Figure 2. *KMT2D* mutation in AML patients.

(A) OncoPrint showing the alternation frequencies of *KMT2D* in AML patients. Data were analyzed from The Cancer Genome Atlas AML project (TCGA-LAML) and OHSU AML cohorts in cBioPortal datasets.

Supplementary Figure 3, related to Figure 3.

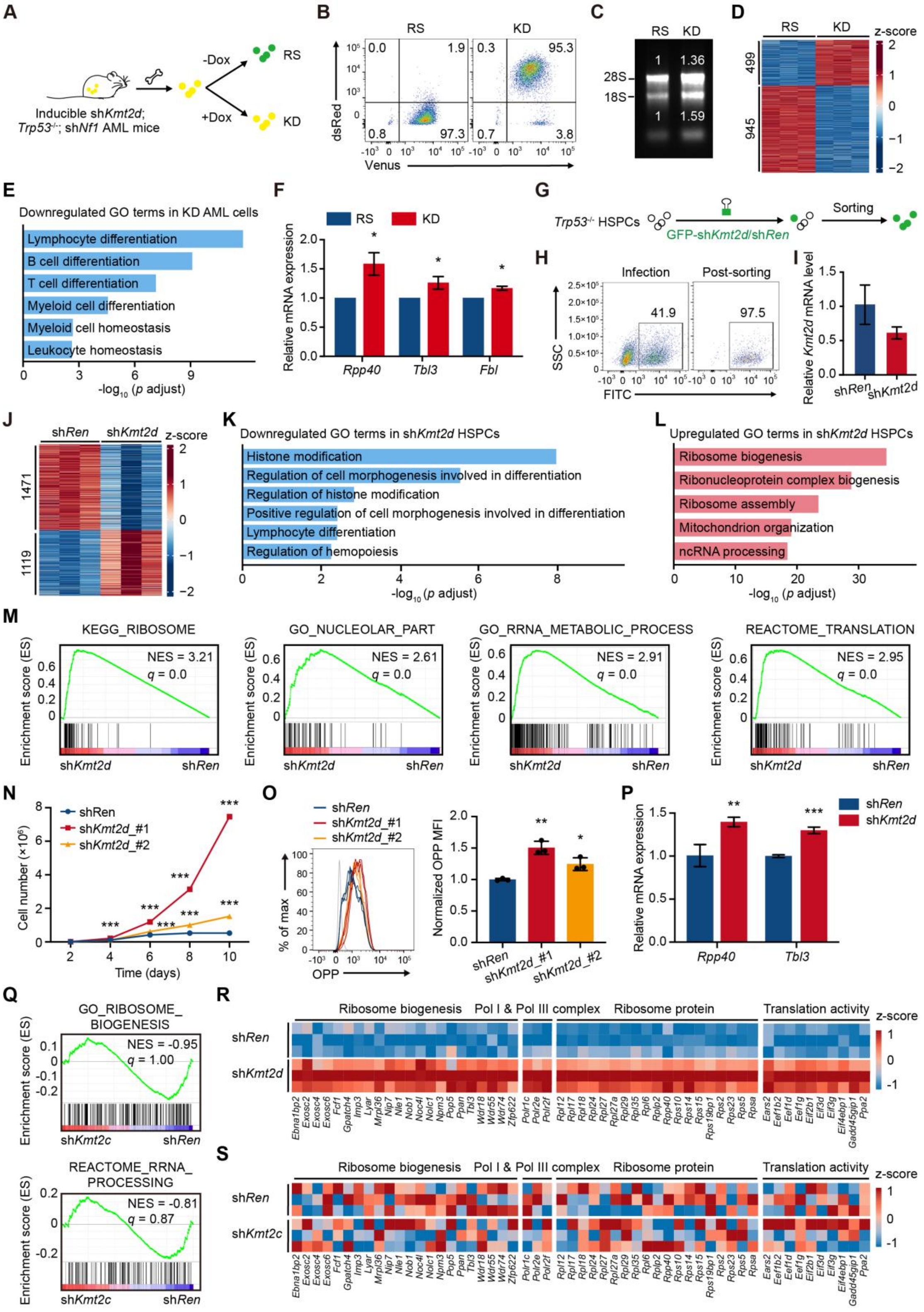

Supplementary Figure 3. *Kmt2d* negatively regulates ribosome biogenesis in AML and HSPCs.

(A) Schematic experimental design for *Kmt2d* restored and knockdown AML cells. Bone

marrow cells from sacrificed TRE-rtTA-driven inducible *Kmt2d* knockdown mice were cultured in vitro. Transcription of dsRed-linked sh*Kmt2d* was turned off (*Kmt2d* restored, RS) in the absence of doxycycline and turned on (*Kmt2d* knockdown, KD) in the presence of doxycycline.

(B) Representative flow cytometry profiles showing the expression of dsRed and Venus in *Kmt2d* restored and knockdown AML cells.

(C) Total RNA from *Kmt2d* restored and knockdown AML cells was separated by non-denaturing agarose gel (1%) electrophoresis. The positions of 18S and 28S ribosomal RNA are indicated. Relative levels of rRNA were quantitated by Image J. Leukemia cells obtained from three inducible sh*Kmt2d*-driven AML mice were tested.

(D) Heatmap showing differential expression genes between *Kmt2d* restored and knockdown AML cells (n = 3 *per* group; log2-fold change > 0.5 or < -0.5; $p < 0.05$, Wald test).

(E) GO analysis of significantly downregulated genes in *Kmt2d* knockdown AML cells compared to restored cells (log2-fold change < -0.5, $p < 0.05$). *P* adjust values were determined by Benjamini-Hochberg correction (*p* adjust < 0.05).

(F) Relative mRNA levels of genes regulating ribosome biogenesis in *Kmt2d* restored and knockdown AML cells were quantified by qRT-PCR (normalized to *Hprt*; n = 3 *per* group).

(G) Schematic experimental design for RNA-seq. *Trp53*$^{-/-}$ mouse HSPCs were transduced with GFP-linked sh*Kmt2d*/sh*Ren*, and then sorted on the basis of GFP fluorescence to >95% purity.

(H) Representative flow cytometric profiles showing the percentages of GFP^+ HSPCs before and after sorting.

(I) Relative mRNA levels of *Kmt2d* in sh*Ren* and sh*Kmt2d* HSPCs were quantified by qRT-PCR (normalized to *Hprt*, n = 3 *per* group).

(J) Heatmap showing differential expression genes between sh*Ren* and sh*Kmt2d* HSPCs (n = 3 *per* group; log2-fold change > 0.5 or < -0.5; $p < 0.05$, Wald test).

(K) GO analysis of significantly downregulated genes in sh*Kmt2d* HSPCs compared to sh*Ren* cells (log2-fold change < -0.5, $p < 0.05$). *P* adjust values were determined by Benjamini-Hochberg correction (*p* adjust < 0.05).

(L) GO analysis of significantly upregulated genes in sh*Kmt2d* HSPCs compared to sh*Ren* cells (log2-fold change > 0.5, $p < 0.05$). *P* adjust values were determined by Benjamini-Hochberg correction (*p* adjust < 0.05).

(M) GSEA showing the positive enrichment of the KEGG_RIBOSOME, GO_NUCLEOLAR_PART, GO_RRNA_METABOLIC_PROCESS and REACTOME_TRANSLATION gene sets in sh*Kmt2d* HSPCs compared to sh*Ren* cells.

(N) The effect of *Kmt2d* knockdown on HSPC growth (n = 3 *per* group).

(O) The effect of *Kmt2d* knockdown on protein synthesis was performed by OPP incorporation assay in sh*Ren* and sh*Kmt2d* HSPCs. Representative flow cytometric profile (left) and quantitation of OPP MFI (right) (n = 3 *per* group).

(P) Relative mRNA levels of genes regulating ribosome biogenesis in sh*Ren* and sh*Kmt2d* HSPCs were quantified by qRT-PCR (normalized to *Hprt*; n = 3 *per* group).

(Q) GSEA showing the enrichment of the GO_RIBOSOME_BIOGENESIS and REACTOME_RRNA_PROCESSING gene sets in sh*Ren* or sh*Kmt2c* HSPCs.

(R) Heatmaps showing differential expression genes involved in ribosome biogenesis, RNA polymerase I & III complexes, ribosomal proteins and translation activity between sh*Ren* and sh*Kmt2d* HSPCs (n = 3 *per* group).

(S) Heatmaps showing differential expression genes involved in ribosome biogenesis, RNA polymerase I & III complexes, ribosomal proteins and translation activity between sh*Ren* and sh*Kmt2c* HSPCs (n = 3 *per* group).

(F), (I), (N), (O) and (P) Graph represents the mean ± SD, * $p < 0.05$, **$p < 0.01$, ***$p < 0.001$ (unpaired two-tailed *t*-test).

Supplementary Figure 4, related to Figure 4.

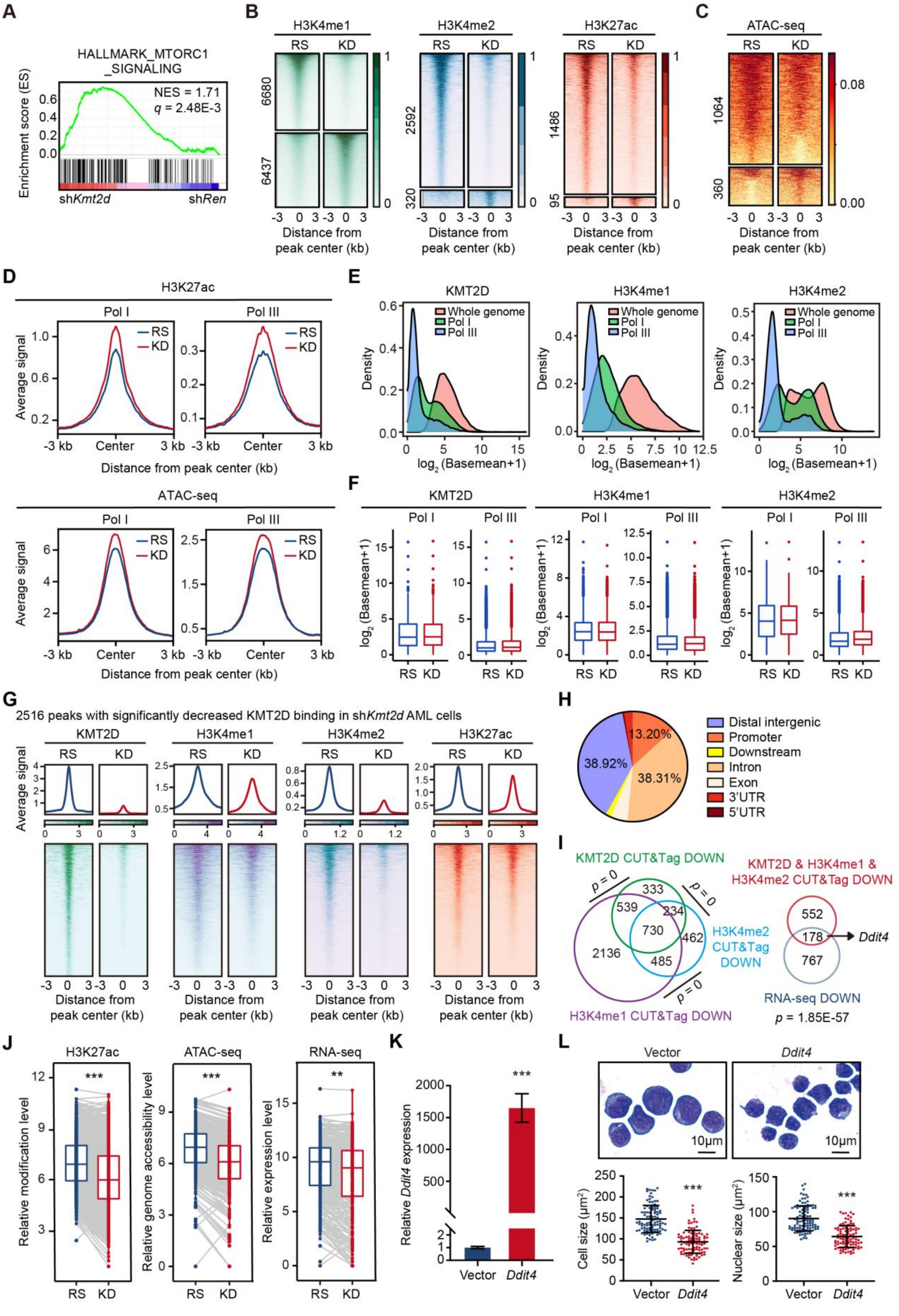

Supplementary Figure 4. *Kmt2d* regulates ribosome biogenesis through histone modification and chromosome accessibility.

(A) GSEA showing the positive enrichment of the HALLMARK_MTORC1_SIGNALING gene set in sh*Kmt2d* HSPCs compared to sh*Ren* cells.

(B) Tornado plots showing differential H3K4me1, H3K4me2 and H3K27ac modification levels between *Kmt2d* restored and knockdown AML cells. Scale bars denote BPM (bins per million mapped reads) for CUT&Tag signal (n = 2 *per* group; absolute log2-fold change > 1; $p < 0.05$, Wald test).

(C) Tornado plots showing differential genome accessibility levels between *Kmt2d* restored and knockdown AML cells (n = 2 *per* group; absolute log2-fold change > 0.5; $p < 0.05$, Wald test).

(D) Average intensity curves showing differential H3K27ac modification levels (top) and genome accessibility levels (bottom) for RNA polymerase I & III binding sites in *Kmt2d* restored and knockdown AML cells.

(E) Density plots showing the KMT2D binding levels, H3K4me1 and H3K4me2 modification levels in the whole genome or RNA polymerase I & III binding sites.

(F) Box plots showing KMT2D binding levels, H3K4me1 and H3K4me2 modification levels in RNA polymerase I & III binding sites between *Kmt2d* knockdown and restored AML cells.

(G) Average intensity curves (top) and tornado plots (bottom) showing 2516 peaks with significantly decreased KMT2D binding in *Kmt2d* knockdown AML cells compared to restored groups. And the H3K4me1, H3K4me2 and H3K27ac modification levels in these corresponding regions between *Kmt2d* restored and knockdown AML cells.

(H) Pie chart showing the distribution of significantly decreased KMT2D binding peaks in

annotated regions of the genome in *Kmt2d* knockdown AML cells compared to restored cells.

(I) Left, Venn diagram showing overlapping of genes with significantly decreased KMT2D binding (n = 1836), H3K4me1 (n = 3890) and H3K4me2 modifications (n = 1911) in *Kmt2d* knockdown AML cells compared to restored cells. Right, Venn diagram showing the overlap between the overlapping genes from the left (n = 730) and genes downregulated in *Kmt2d* knockdown AML cells compared to restored cells (n = 945) . *P* values were determined by hypergeometric distribution.

(J) Box plots showing differential peaks of H3K27ac modification, genome accessibility and expression levels of overlapping genes in (I) (n = 730); ***p* < 0.01, ****p* < 0.001 (two-tailed Wilcoxon rank-sum test).

(K) Relative mRNA levels of *Ddit4* in *Kmt2d* knockdown AML cells transduced with vector or *Ddit4* cDNA were quantified by qRT-PCR (normalized to *Hprt*, n = 3 *per* group). Graph represents the mean ± SD, ****p* < 0.001 (unpaired two-tailed *t*-test).

(L) The effect of *Ddit4* overexpression on cell morphology. Representative pictures performed on Liu's-stained cytospins (top) and quantitation of cell and nuclear size (bottom) (n = 100 *per* group).

Supplementary Figure 5, related to Figure 5.

A

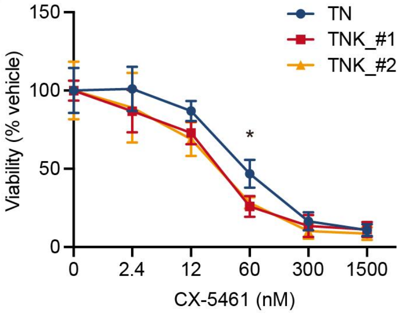

Supplementary Figure 5. *Kmt2d*-deficient AML cells are sensitive to CX-5461 treatment.

(A) Leukemia cells harvested from TN (*Trp53*$^{-/-}$; sh*Nf1*; sh*Ren*) or TNK (*Trp53*$^{-/-}$; sh*Nf1*; sh*Kmt2d*) mice were incubated with various concentration of CX-5461 for 72 hours. Graph represents the mean ± SD (n = 3 *per* group); $^{*}p < 0.05$ (unpaired two-tailed *t*-test).

Supplementary Figure 6, related to Figure 6.

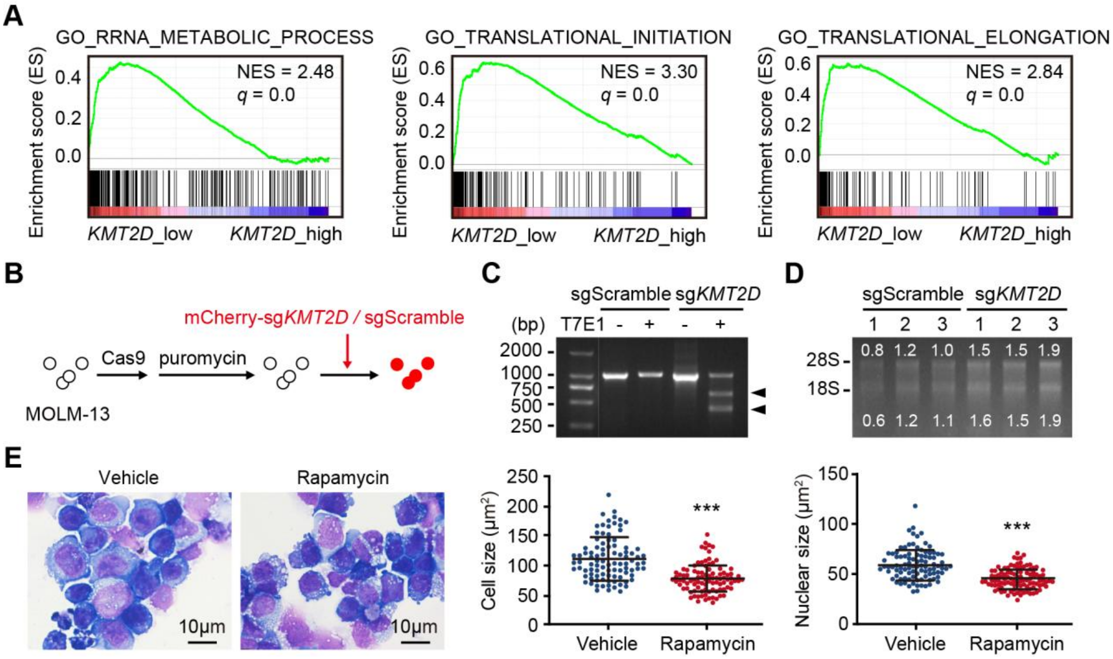

Supplementary Figure 6. ***KMT2D*** **regulates ribosome biogenesis in human AML.**

(A) GSEA showing the positive enrichment of the GO_RRNA_METABOLIC_PROCESS gene set, the GO_TRANSLATIONAL_INITIATION gene set and the GO_TRANSLATIONAL_ELONGATION gene set in *KMT2D* low expression AML patients (n = 20) compared to *KMT2D* high expression ones (n = 122) in the TCGA-LAML cohort.

(B) Schematic experimental design for *KMT2D*-mutated MOLM-13 cells. MOLM-13 cell lines were transduced with *Cas9*, selected with puromycin, and then transduced with mCherry-linked sg*Kmt2d* or sgScramble.

(C) T7 endonuclease I assay on *KMT2D* in MOLM-13 cell lines.

(D) Total RNA from MOLM-13 cell lines with sgScramble or sg*KMT2D* was separated by non-denaturing agarose gel (1%) electrophoresis. The positions of 18S and 28S ribosomal RNA are indicated. Relative levels of rRNA were quantitated by Image J. Leukemia cells obtained from three inducible sh*Kmt2d*-driven AML mice were tested.

(E) The effect of rapamycin on cell morphology. Representative pictures performed on Liu’s-stained cytospins (left) and quantitation of cell size and nuclear size (right) of rapamycin-treated and vehicle-treated sg*KMT2D* MOLM-13 cells (n = 90 *per* group). Graph represents the mean ± SD, ***$p < 0.001$ (unpaired two-tailed *t*-test).
